# Sustainability of Evidence-Based Practices for HIV Prevention among Female Sex Workers in Mexico

**DOI:** 10.1371/journal.pone.0141508

**Published:** 2015-10-30

**Authors:** Lawrence A. Palinkas, Claudia V. Chavarin, Claudia M. Rafful, Mee Young Um, Doroteo V. Mendoza, Hugo Staines, Gregory A. Aarons, Thomas L. Patterson

**Affiliations:** 1 School of Social Work, University of Southern California, Los Angeles, CA, United States of America; 2 Department of Psychiatry, School of Medicine, University of California San Diego, La Jolla, CA, United States of America; 3 Division of Global Public Health, School of Medicine, University of California San Diego, La Jolla, CA, United States of America; 4 School of Public Health, San Diego State University, San Diego, CA, United States of America; 5 Research and Evaluation Unit, Mexican Foundation for Family Planning (Mexfam), Mexico City, Mexico; 6 Faculty of Biomedical Sciences, Autonomous University of Ciudad Juarez, Ciudad Juárez, Chihuahua, Mexico; University of Malaya, MALAYSIA

## Abstract

**Objective:**

This study examined service provider perceptions of requirements for successful sustainment of an efficacious intervention for preventing HIV/AIDS and STIs in female sex workers (FSWs) in Mexico.

**Methods:**

Semi-structured interviews were conducted with 77 leaders and counselors from 12 community-based reproductive health clinics located throughout Mexico participating in a large hybrid effectiveness-implementation randomized controlled trial to scale-up the use of Mujer Segura, a psychoeducational intervention designed to promote condom use and enhance safer sex negotiation skills among FSWs.

**Results:**

Five sets of requirements for sustainment were identified: 1) characteristics of the provider, including competence in delivering the intervention, need for continued technical support and assistance from outside experts, and satisfaction with addressing the needs of this population; 2) characteristics of the clients (i.e., FSWs), including client need and demand for services and incentives for participation; 3) characteristics of the organization, including its mission, benefits, and operations; 4) characteristics of the outer setting, including financial support and relationship with the community-based organization’s central offices, and transportation and security in areas where FSWs live and work; and 5) outcomes associated with the intervention itself, including a reduction of risk through education and increased outreach through referrals from FSWs who received the intervention.

**Conclusions:**

Although the requirements for successful sustainment of interventions like Mujer Segura are consistent with the factors identified in many models of implementation, the results illustrate the importance of local context in assigning priority to these model elements and suggest that the five categories are not discrete entities but interconnected.

## Introduction

According to UNAIDS [1], there were an estimated 36.9 million people worldwide living with HIV/AIDS at the end of 2014, an increase of approximately 2 million people from the previous year. The great majority of these people live in low- and middle-income countries (LMICs) [1]. Global HIV prevention efforts have focused on various risk populations, including female sex workers (FSWs), men who have sex with men (MSMs), and people who inject drugs (PWID). These efforts have included the implementation of low-cost, culturally appropriate, evidence-based interventions, including test and treat (T&T) [2,3], pre-exposure prophylaxis (PrEP) [4–6], community empowerment [7,8], and psychoeducational programs designed to promote condom use in men and women [9–11].

However, successful implementation and sustainment of these efforts in LMICs remains limited [12–17], in part, due to the lack of information as to the requirements for successful implementation and sustainment [18–21]. For the most part, characteristics of the outer (e.g., policy, funding, etc.) and internal (i.e., organization, providers, etc.) contexts or settings, providers, clients, and the intervention itself, that are central components of most implementation models [22–25] have been unexamined. Many of these models consider sustainment to be the final stage of the process of implementation [22,25], but the factors that predict sustainment are not well understood [26,27]. In part, this may be attributed to a lack of consensus as to what constitutes sustainment and how to measure it. There are no uniform or agreed upon criteria for determining whether something has been sustained or not, but a recent review offers some guidance regarding levels of sustainment [28].

A recently completed investigation of the implementation of an intervention for prevention of HIV and STDs among FSWs in Mexico provides an opportunity to examine these factors. Mujer Segura (“Healthy Woman”) is an efficacious behavioral intervention for Mexican FSWs, which used motivational interviewing and behavior change principles (i.e., social cognitive theory) to increase condom use and safer sex negotiation skills with clients. The current standard of care for HIV prevention with FSWs in Mexico is set by the Centro Nacional para la prevención y el Control del VIH/SIDA (CENSIDA) and consists of pre- post text counseling. Currently this counseling is lecture-based and does not utilize theory-based methods of behavior change used by Mujer Segura. An earlier study found that FSWs randomized into Mujer Segura had a 40% decline in cumulative incidence of sexually transmitted infections (STIs), with significant decreases in sexually transmitted infections and HIV, and concomitant increases in total numbers and percentages of protected sex acts and decreases in total numbers of unprotected sex acts with clients at six-month follow-up [29]. Partnering with the Mexican Foundation for Family Planning (MexFam), a non-governmental organization (NGO) that has sites throughout Mexico, we examined whether a “train the trainer” model of implementation [30,31] could develop a network of HIV/STI prevention services for FSWs with self-sustaining levels of model fidelity and provider competency. The specific aims of the parent study were to: 1) determine if our implementation model could achieve high levels of intervention fidelity and provider competency in the context of a large-scale implementation effort; 2) characterize the relationship between individual provider characteristics and organizational factors and determine their impact on the implementation of Mujer Segura using a mixed-methods (quantitative and qualitative) approach; 3) determine whether the implementation of Mujer Segura by community-based organizations (CBOs) is associated with decreased sexual risk behaviors among Mexican FSWs over a six-month period (e.g., increased condom use with clients, reductions in STI incidence); and 4) determine whether improvements in sexual risk behaviors among Mujer Segura FSWs are associated with variations in intervention fidelity and counselor competency.

One of the goals of Mujer Segura was to develop local and culturally relevant expertise and infrastructure to sustain and further disseminate the Mujer Segura intervention upon completion of this project. The aim of this study was to identify requirements for sustainment of Mujer Segura as perceived by study participants. As there is no widely accepted model of evidence based practice (EBP) sustainment in LMICs like Mexico, we explored the issue using qualitative methods [14,32].

## Methods

### Design

Mujer Segura was a multi-site, randomized controlled trial (RCT), with a two-arm, wait-list design and a 50/50 allocation ratio of sex workers that tested a safer-sex intervention for FSWs using a “train the trainer” model of implementation. Also known as Mujer Segura, the clinical intervention is a brief (35 to 40 minutes), single-session, intervention that combines principles of motivational interviewing (MI), social cognitive theory (SCT), and the theory of reasoned action [33,34]. The counselor uses MI techniques (e.g., key questions, reflective listening, summarization, affirmation, and appropriate use of cultural cues) to increase the participant’s motivations to practice safer sex. In the context of sexual risk reduction counseling, “train-the-trainer” involves identifying a staff member who has some expertise in HIV/STI counseling and teaching that person how to train other staff in delivery of the counseling program. The “train the trainer” implementation strategy is considered a good choice for agencies with limited financial resources, such as CBOs and non-profit organizations [35,36]. Complete details of the intervention and the RCT are found elsewhere [28,37]. The present study focuses on qualitative interviews conducted with staff at each site upon completion of the intervention.

### Participants

The CBOs chosen for this implementation study were all part of MexFam, a non-profit NGO. Headquartered in Mexico City, MexFam operates sexual and reproductive health programs in 22 states in Mexico. Among its other community health programs, MexFam has worked to increase HIV prevention through advocacy, gender- and culture sensitive interventions, and educational media campaigns [38]. To make the sample of sites representative, MexFam included CBOs with varying capacities, sizes, and geographic locations. Each site had to meet the following minimum criteria: a staff member qualified to be an internal trainer; a core of approximately six to eight high-potential, stable staff members who could be trained as intervention counselors; an organizational culture that supports innovation and evidence-based perspectives; a strong positive reputation in its community; the capacity to deliver professional peer-to-peer training; and strong cultural competency and willingness to work with FSWs. From 23 eligible clinics, 13 were randomly drawn for participation in this study. One site subsequently withdrew from the study, leaving 12 sites at the time of follow-up. Participants from each clinic included one internal trainer, approximately six to eight persons qualified to deliver the intervention, two supervisors, and the local CBO administrator. Of the 93 participants who completed the battery of questionnaires at the follow-up visit, 76 percent were women, the average age was 35 (S.D. = 10.7) years, and the average number of years of formal education was 1.6 (S.D. = 3.5). Of these participants, 77 (82.8%) also completed a semi-structured interview.

This study, including procedures for obtaining informed consent, was approved by Institutional Review Boards in the United States and Mexico prior to participant recruitment. Written informed consent was obtained from study participants prior to data collection; all consents are on file.

### Implementation process

Implementation of the “train-the-trainer” approach proceeded through four phases. In phase one, selected CBO staff responded to questionnaires measuring provider and organizational factors and participated in qualitative interviews and focus groups conducted by ethnographers. CBO outreach workers subsequently recruited 80 FSWs to participate in the effectiveness trial. FSWs assigned to the standard care condition were assessed and received their intervention. In phase two, a CBO staff member was selected to become the organization's ‘internal trainer.’ This person received intensive training in the Mujer Segura intervention by the developers of the model and by ‘practice experts’ (counselors who delivered Mujer Segura in our earlier study). Internal trainers practiced delivering the intervention while being coached in vivo by the model developers and practice experts until achieving formal certification of proficiency. In phase three, additional CBO staff persons received Mujer Segura training from the internal trainer. When CBO counselor trainees met criteria for practice competency, outreach workers administered baseline behavioral assessments to the FSWs randomly assigned to Mujer Segura, after which the trained CBO counselors began to deliver the intervention. Fidelity to the intervention protocol was monitored through direct observation by the internal trainer and completion of fidelity checklists by both FSW participants and the counselors themselves. In phase four, internal trainers shifted to a maintenance function, which included training and coaching new hires and a less-intensive schedule of in vivo coaching and monitoring of counselors’ fidelity to the intervention model.

### Data collection

Data for this present study was obtained from follow-up semi-structured interviews with 77 CBO leaders, supervisors and counselors participating in the RCT. Each interview was conducted in Spanish and lasted approximately 60 minutes. These interviews addressed a subset of organizational characteristics defined as operationally salient in the implementation model. Questions in the follow-up interview elicited information on knowledge, attitudes, and behavior related to the intervention and characteristics of the implementation process. Questions were sufficiently open-ended to allow respondents to elaborate on issues they consider important or relevant. Sample questions included: How prepared do you feel you are to continue delivering this intervention without the involvement of the researchers? What would make you more prepared? What type of assistance do you think will be needed from the researchers to sustain Mujer Segura in your CBO? To what extent do you support the continued delivery of Mujer Segura in your CBO? Interviews were audio-recorded and transcribed for analysis.

We also conducted focus groups with study participants in an effort to confirm the results from our qualitative analyses of the semi-structured interviews. While the results reported in this manuscript are based on the semi-structured interviews only, we only reported those results that were confirmed by the focus groups as being accurate.

### Data Analysis

A methodology of “Coding Consensus, Co-occurrence, and Comparison” [39] rooted in Grounded Theory (i.e., theory derived from data and then illustrated by characteristic examples of data) [40] was used to analyze semi-structured interviews. Audio-recorded interviews were transcribed and reviewed by three investigators, who individually developed and applied a series of “open” codes to describe the information contained in the transcripts [41]. These codes were subsequently discussed, matched and then integrated into a single codebook. Each text was independently coded by at least two investigators and disagreements in assignment or description of codes was resolved through discussion between investigators and enhanced definition of codes. The final list of codes, or codebook, constructed through a consensus of team members, consisted of a numbered list of themes, issues, accounts of behaviors, and opinions that related to individual, organizational and system characteristics that influence sustainment of Mujer Segura. Inter-rater reliability in the assignment of specific codes to specific transcript segments was assessed for a subset of randomly selected pages from 10 transcripts. For all coded text statements, the coders agreed on the codes 98% (range = 96% - 100%) of the time, indicating good reliability in qualitative research [42]. The computer program Dedoose [43] was used for coding and generating a series of categories arranged in a treelike structure connecting text segments as separate categories of codes or “nodes.” Aggregations of codes through a process of constant comparison were used to construct themes and subthemes. The percentage of participants who identified a specific requirement for sustainability, stratified by role in the organization (counselors versus leaders) was used to illustrate their importance or salience. These nodes and trees were used to further the process of axial and pattern coding [41] to examine the association between different a priori and emergent categories related to the topic of sustainability of Mujer Segura. English translations of text segments were reviewed by bilingual investigators for accuracy.

## Results

**Fig 1:**
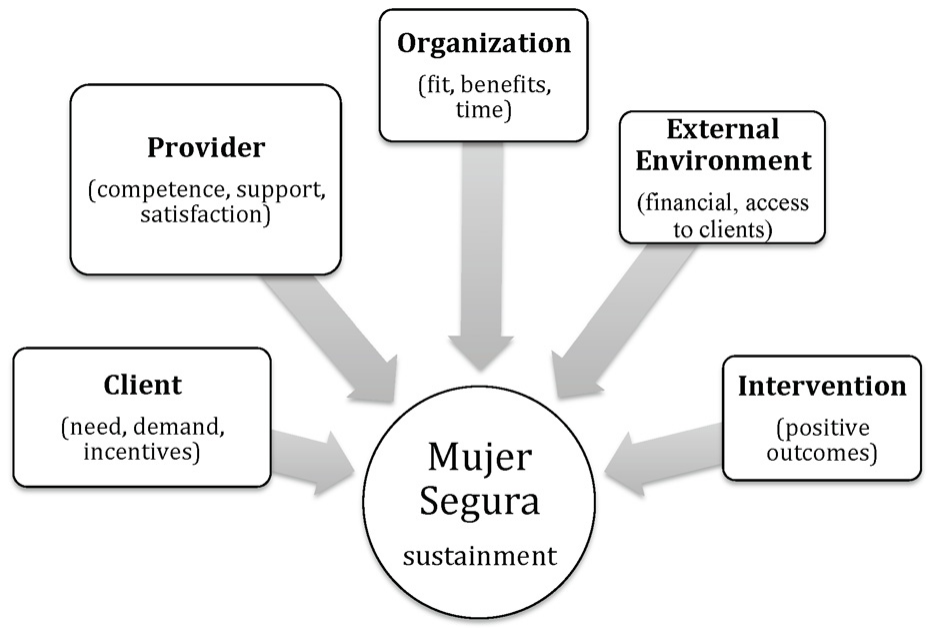
Requirements for sustainment of the Mujer Segura intervention.

Analysis of the qualitative data obtained from the semi-structured interviews revealed five sets of characteristics perceived to be associated with successful sustainment of the Mujer Segura intervention: provider, client, organizational, external environment, and outcomes. These characteristics were framed by study participants as requirements and/or reasons for sustaining the intervention; in a few instances, they were framed as challenges to be overcome. Fig 1 below provides an illustration of the groups and subgroups of characteristics with each group of characteristics sized (i.e., weighted) by percent of participants who identified it as a requirement for sustainment. Each of these sets is examined in detail below.

### Provider Characteristics

All of the counselors were asked how prepared they were to continue delivering the intervention without the involvement of the researchers, and 85.2% of the counselors and 85.7% of the leaders (85.3% total) reported they were prepared and felt competent to deliver the intervention. However, as the following statement illustrates, perceptions of competence in delivering the intervention were both linked to a desire to continue receiving feedback from the research investigators and to feelings of satisfaction associated with the activity itself.

“Prepared? Yes I feel prepared to continue with the model that I have, right? With the experience that I have, but just to see if the counseling that I was applying was good, I would like feedback… I think that it was good, but then perhaps you as investigators may think that my implementation was not the correct one. But I do feel prepared, I liked it, I liked working with this population. I like working with this population” (counselor).

Continued technical assistance from the researchers was considered by 63.0% of counselors and 57.1% of leaders (67.6% total) to be an important requirement for sustainment of the intervention. One supervisor felt that the training provided by the researchers was adequate and did not require any additional support: “Not for counseling. I think that the training that was given to the person who was to replicate the counseling was very good and the training she gave to the other people of the center was good, everything was clear.” However, most of the counselors expressed a preference for some form of ongoing feedback, advice, updates, and monitoring of their performance in using the intervention. According to one counselor:

“I think that from now on, maybe having at some time, maybe a little assessing, maybe at a distance with the technical support that we have now, I think that we can continue implementing the activities within the risk group and in this clinic. … Maybe some continuous training or the support with technical manuals, or maybe some written information that continues giving us feedback in some way when it comes to the benefits and the reach of the project that it had once it was implemented and the results of the protocol were given.”

Such technical support was viewed as a critical element of provider competence in using the intervention and, as noted by another counselor, a source of reassurance in those instances when unanticipated circumstances or questions arise: “Well, for example, what has worked for me with other specific groups is that someone is always there when you have a doubt you can ask and get an answer quickly, this assistance is to me like a high priority” (counselor).

Provider satisfaction was a common theme in 25.9% of the counselor interviews as well. Several of the counselors reported that they enjoyed working with this population and they developed a special bond in working with them. Some counselors acknowledged personal gains from having provided the service: “Of course, I am a person that will support the things we learned from the program, because all in all I had an opportunity to share with them [FSWs] many things that made me more humane, that made me understand them.” In other instances, the personal satisfaction arose from the perception that these interactions resulted in benefits to individual FSWs in the form of increased self-esteem and self-efficacy and to the community in the form of STI prevention.

“Well maybe because of the experiences that I lived with the participants, in a way you appreciate them and, well, they form part of your daily life. For them to trust you to say things that are so personal. You may try to stay within the margin and not, get too involved, but they are people all those women, are outcasts, they are distant. Like they say, “we are the plague,” and I say, “no, no, not at all”. So then you give them condoms and it does help them, and if you can do it well, that is cool. So the appreciation you gain for them or that you already have for them, and will continue to like help them so that we can stop the infection chain” (counselor)

### Client Characteristics

The desire to meet client needs was identified by 59.2% of the counselors and 57.1% of the leaders (58.8% total) as a reason for sustaining the Mujer Segura intervention. A little over 40% of the counselors and 42.9% of the leaders noted that their communities in general and FSWs in particular were in need of services to prevent HIV and STIs. This need was attributed largely to a lack of knowledge or understanding on the part of the FSWs as to the nature of the infections, their consequences, and methods for prevention and treatment.

“There are people that truly don’t know how big the illness is. I spoke with some and they told me they thought syphilis was like the common cold, that a little pimple appeared and then went away, then the illness also went away, meaning they don’t know that when that little pimple is gone, it is the beginning of a more serious phase of the illness. It is not that is going to go away, meaning they really need to know a lot more. … And it is sad to see a person that can lose their life due to ignorance. If we are in a position to help them, then why not?” (leader).

Counselors also acknowledged they had a certain responsibility to continue providing the service to their existing clients as a reason for sustaining the intervention. As one counselor observed,

"Well, I think one of the main things would be like not leaving the girls who are already in this project, because they can very well take part as community leaders, the ones that are already in, and through them reach other girls, and not only other girls but other people, the impact they are generating as peers."

However, there was also the perception that the intervention could benefit women who were not FSWs and men as well:

“I would support it [i.e., continuing the project] because I think that there are not only certain women that are sexual workers who need it. I think there are others, as they have commented themselves, that there were other women that did want the project to continue now if it could continue to assist in preventing the spread of disease” (counselor).

“Well, we have a person with the knowledge, it might be as part of the follow-up support, as part of the monitoring and feedback to also work a little more with men, because women are trained, but many times it is necessary to involve the man, so as to care for their health, which is what we need to work more, a project is implemented but for men” (director).

Another reason for continuing the program according to counselors is client demand for the intervention. This was identified by 18.5% of the counselors only as a requirement for sustaining the intervention. According to one counselor:

“I would be willing to continue working because… where I go to, I continue seeing them and they continue asking me, they continue telling me ‘hey [name of counselor], when will there be another project for us?’ And I say “‘or now there are none’, but I still have a lot of contact with them practically, so my job hasn’t finished, when they have any doubts, when I have to refer them to a service, there is still a lot of contact with them.”

One of the potential obstacles to sustaining delivery of the service, however, was the potential elimination of financial incentives used during the RCT to encourage FSW participation. Some participants stated there was no longer a need for such incentives.

“I think it was at first a very good strategy for recruitment, but I think right now it would not be as necessary because they know us and they come back. We have seen [they] are coming even with their partners, with their children, or they bring a friend. This has indeed happened, and similar situations without giving them vouchers or samples” (director).

However, 14.8% of the counselors and 14.3% of the leaders expressed concern that without any financial support, clients might be disinclined to visit the clinic for other services as well, including HIV testing:

“According to my view, it would not be hard for me to continue with the counseling, but well but there are many of them [FSWs] who don’t come simply for counseling. Many of them, if you give them something in return, they feel a little more obligated to come for that which you are giving them. But for them to come without giving them anything, it is a little more difficult” (counselor).

In turn, offering incentives for FSWs to come to the clinic was viewed as having an additional benefit of expanding the impact and reach of the Mujer Segura intervention.

“The free test was, in reality, the economic incentive, which were the vouchers that helped them, even though it occurred during only two visits. Suddenly, they would come to get tested for HIV or they would bring friends, saying ‘hey can you do it to someone else?’ Of course, because of the project, we have free tests, and so we would get them that way because what really interests them are the tests, because they are at risk, they worry” (counselor).

### Organizational Characteristics

While the appeal of financial incentives may be viewed as a client characteristic, the ability to provide such incentives may also be viewed as a characteristic of the CBO. Study participants (37% of counselors and 43% of leaders, 38% total) noted other factors that reflected organizational characteristics, such as the consistency of the intervention with the mission of the CBO. According to one of the supervisors:

“The philosophy of this organization was traditionally focused on family planning and birth control for women. In some ways, our programs were not implemented much in these high risk and vulnerable groups within our municipality. I think that it is feasible in the end because it is part of the philosophy and the vision of this organization, but in that in some way it can count on the money, the resources.”

Another factor identified by 11.1% of the counselors and 14.3% of leaders was the benefits to the organization that resulted from the intervention. These benefits included an enhancement of the clinic’s reputation and likelihood of continued support from MexFam because there were no other places in Mexico where such an intervention was currently in use. The intervention also enabled MexFam to fulfill its public obligations.

Another requirement identified by 7.4% of counselors only for sustaining the intervention was having sufficient time to implement it. Some counselors stated that at the present, there was little time available to work with the FSWs given their other responsibilities:

“Well, I think that personally I would be missing a little bit of what was Mujer Segura, maybe I missed studying it more and processing the information, but then you see sometimes we have two projects. The work was a little bit difficult right now, and it added up to a lot [of work] because in the other project that we have [we] are “selling” (i.e., introducing) [contraceptive] methods, and the truth is that it was harder, but it was a little bit more time that was devoted [to Mujer Segura]” (counselor).

To address this issue, another counselor recommended that the team should focus exclusively on one task and not work on others:

“All of my colleagues had other things to do, other tasks to do. We had to go with *promotores* or give talks that we would ease up in performing certain tasks and were being less rigorous with all programs. And so, we reached none of our goals [in the other programs] because the workdays [in Mujer Segura] were very extensive.”

### External Environmental Characteristics

External environmental characteristics were identified by 33.3% of counselors and 57.1% of leaders (38.2% total) as an important requirement for sustainability of the Mujer Segura intervention. Perhaps the most important characteristic of the CBO’s external environment or outer context/setting was the availability of funds necessary for the intervention itself and the other free and low cost services that helped to induce FSWs to attend the clinics. As one counselor noted, financial assistance from the Centro de Salud (local clinic) was essential “because it (i.e., Mujer Segura) really is an expensive program, yes?” However, while some participants stated that MexFam has always provided support and advice when needed, other participants expressed the opinion that successful sustainment required a certain degree of independence from the MexFam central offices in Mexico City and greater reliance on local resources. According to one CBO director, “The issues of materials and supplies so often depend on the funding, but many times one can get some things locally, like donations from the health sector. Then, you are betting that the project can continue to some degree and work can benefit from a donation by the Secretariat of Health.” Another counselor noted:

“Regardless of what happens in Mexico City [i.e., MexFam Central Office], the project here is alive, and even without Mexico City, we are going to continue… because when we started with the community programs in this clinic, we were without financing for two years and we managed to keep afloat without help from the central office…. And currently, we depend on help from Mexico City, but I think now with more tools and with this team, it would be easier even if they told us that they can no longer support us, I feel that we can be self-sufficient.”

Regardless of the source, there was a general belief that sustainment of the program required financial support, in part, to offer free or low cost services as an incentive, also noted earlier.

Another requirement identified by 11.1% of counselors only concerned the need to provide counselors with transportation and security when traveling to neighborhoods where FSWs were likely to be found. Several participants commented on the level of criminal activity and violence in the “zonas rojas” where FSWs were likely to live and work.

“Well, I think [that to continue implementing Mujer Segura] we would need a means of transportation, because, for example, we needed to go to those places with our own car or because we had to walk from one side [of town] to another or to get into [the sites], and be more secure in returning [to the agency], and when leaving [the zona rosa], make it faster, because yes, there is always someone who can generate violence in the community" (counselor).

“Well I think that in general the stigma we have with the population itself hinders a little the recruiting process because, for example we had women in [a factory district close to Mexico City], that are regulated, so to speak; then there was much reluctance by managers (i.e., pimps) that work with them or maybe it could be seen as part of something that might affect them and on the other hand, openly recruiting in the streets, or look for places where we could recruit, was complicated and also risky for the recruiters” (counselor).

### Intervention Characteristics

The final requirement for sustaining Mujer Segura as reported by 37% of counselors and 14.3% of leaders (32.4% total) was the observation of positive outcomes. For instance, when asked if she would advocate for the continued use of Mujer Segura once the study came to an end, one counselor stated: “aside from also giving us prestige here at the clinic, we are assisting this type of population to take care of themselves, and more than anything, to avoid sexually transmitted diseases with the information that is being given to them.” Even though the study results had yet to be published, counselors were convinced the impact was positive. According to one such participant, “I am fully convinced that it is part of our mission, the results are very good and it's really great to have opportunities to work with such groups that are so vulnerable.” Another counselor concluded: “Even if there wasn’t any support, we would continue implementing it because we have observed the process very closely, and this has enabled us to see the results.” However, the benefits were also seen to extend to the general community, as explained by one of the supervisors:

“Well, one of the reasons [for sustaining Mujer Segura] is the benefit that it leaves the community where I interact. It improves the quality of health of certain vulnerable groups that are in a certain way outcast and that are deprived of access to support programs or social benefits, and therefore become involved in other types of demeaning situations or relegated by certain activities that some people see as in a very particular manner because of their religious beliefs as something they deserve. But the health benefit in general for this municipality is very high, and I think that is part of the factors that allow me to see the necessity for this to continue to be implemented” (supervisor).

These benefits, in turn, were viewed as outweighing any potential cost to the client and to the organization.

“Those people [FSWs] don’t come and then seek medical attention or detection, because they know it is going to generate a cost and financial burden for them. But, if the project continues, if part of the activities performed as part of the project would remain permanently, and it would be made known to them that it wouldn’t generate any personal cost, it would be of great benefit to them and for the community because they are at risk of infection and most of the time, they lack the awareness. Or, when we have suggested the use of the condom, a lot of the time we don’t know whether they use it at all, or if they used it in a regular manner. Well, it could impact their health in a very important way” (supervisor).

## Discussion

In this study, we identified five sets of requirements for sustainment in individual-level HIV prevention interventions in a LMIC like Mexico. The first set of requirements was comprised of characteristics of the provider and included perceptions of competence in delivering the intervention, need for continued technical support and assistance from outside experts, and satisfaction with addressing the needs of this population. The second set of requirements was comprised of characteristics of the clients (i.e., FSWs), including need and demand for services and incentives for participation. The third set of requirements was comprised of characteristics of the organization (i.e., the CBO) and included its mission (a part of the organizational culture), benefits, financial support, and operations (i.e., minimizing provider work overload). The fourth set of requirements was comprised of characteristics of the outer setting and included financial support and relationships with the CBO central offices in Mexico City and the ability to overcome logistical and safety constraints associated with outreach efforts and client access in neighborhoods where FSWs lived and worked. The final set included the outcomes associated with the intervention itself. These outcomes included a reduction of risk through education and increased outreach through referrals from FSWs who received the intervention. Although we narrowed our focus to one specific risk population in one specific LMIC, our findings could generalize not only to future implementation efforts involving Mujer Segura but also to the implementation of other evidence-based interventions in resource-constrained settings.

Many of these requirements have been identified in previous studies of HIV prevention in LMICs, including client demand for services, access to financing and resources subsequent to the transition from research to routine practice, and increased outreach to potential clients [2,17]. Other requirements identified in previous studies were not mentioned or prominently featured. For instance, there was no mention of the political will required to provide services to FSWs [2,17], although one counselor did comment on the challenge of delivering services to women whom some believed, for religious reasons, did not deserve access to such services. However, the participants’ perspectives were focused on the clinic and personal experiences with implementation, which is consistent with their organizational roles. The focus of comments relating to financial support was limited to discussions of purchasing of condoms and other supplies and making other services available for free or low cost so as to encourage FSWs to come into the clinic and make themselves available for receiving the intervention. The stigma attached to both HIV and sex work, frequently noted in earlier research [15–17], was also barely mentioned by counselors as a barrier to sustainment. Rather, as noted above, there was a certain degree of satisfaction in providing to women who referred to themselves as “the plague” a certain measure of self efficacy and self worth.

In many respects, the requirements for successful sustainment of interventions like Mujer Segura are consistent with the factors identified in most of the current models of implementation. For instance, concern about funding and financial support and the relationship with MexFam’s central office in Mexico City reflect elements of the environment [25] or “outer context” of implementation [22,23]. The mission or organizational culture of the CBO that prescribes delivering services to FSWs, benefits to the organization’s reputation, and measures to prevent work overload of counselors reflect elements of the organization [25] or “inner context” [22,23]. This third set is consistent with recommendations for using multiple strategies to develop a positive implementation climate through the use of “embedding mechanisms” to support effective implementation and sustainment of evidence-based practices within health systems and organizations [44]. Counselor perceptions of competence in using the intervention, motivation for doing so (i.e., satisfaction and feelings of accomplishment), and needs for ongoing technical assistance, transportation and security reflect elements of provider characteristics. Client demand and availability reflect characteristics of the client, and the positive outcomes associated with the intervention itself reflect an important characteristic of the intervention and its fit for the organization, providers, and clients [22,23]. While the Mujer Segura intervention has not been tested with other populations such as high risk youth, there are commonalities between young and older FSWs such as the need for knowledge, negotiation skills etc. However, there are unique challenges faced by FSW youth including the fact that about 25–50% of FSWs who enter sex work were trafficked before the age of 18 [45, 46]. In addition, sex work in Mexico is illegal for women under the age of 18, making them difficult to reach. These and similar issues suggest the need to modify and test Mujer Segura in younger sex workers.

The five sets of characteristics also exhibit a certain degree of correspondence with a new behavioral model of implementation [47]. This model identifies three primary concerns of providers when considering the adoption of new and innovative programs: costs and benefits, capacity, and acceptability. In this study, costs were represented in terms of purchase of condoms and providing free or low cost services as an inducement for FSWs to receive the Mujer Segura intervention, while benefits were represented in terms of reducing HIV and STI prevalence in the community and enhancement of the organization’s reputation for delivering services. Capacity was represented in the form of comments regarding counselor competence in delivering the intervention, need for technical assistance from external experts, provision of transportation and security, availability of low cost or free services, availability of funding to support services delivery, and the need to prevent counselor workload. Acceptability was represented in terms of provider satisfaction and FSWs’ need and demand for services.

However, the findings from this study provide additional insight as to the relevance of these models for understanding sustainment of evidence-based and innovative practices in LMICs. First, they illustrate the importance of local context in assigning priority to these model elements. Every study participant stated that they were confident in their being able to continue delivering the intervention because of the training they had received, but almost every participant also acknowledged the value and importance of continued technical assistance in the form of feedback, advice, updates and fidelity monitoring. These two requirements represent a tension between desired autonomy and self-reliance needed to deliver services and reliance upon external experts, usually from high-income countries, for support. Study participants were especially appreciative of the training they received because of their limited experience in being trained in EBPs. A similar tension was observed between continued reliance upon the central offices of MexFam and the perceived need to utilize local resources. Participants acknowledged the importance of continued financial support, but not all sites were confident that such support from MexFam would be forthcoming or that it was even desired. Local context is also relevant with respect to outreach efforts in parts of the community where personal safety and provider access are concerns, and to the satisfaction gained from and perceived need for reaching out to and interacting with a traditionally underserved and stigmatized population.

Second, the findings point to the conclusion that the five categories illustrated in Fig 1 are not discrete entities but interconnected. Competence in delivering the intervention (a provider characteristic), the clinic’s ability to provide sufficient time to deliver the intervention and incentives for FSWs to visit the clinic (organizational characteristics), the client demand for the service (a client characteristic), and MexFam’s ability to provide continued funding to support the intervention and access to clients (an environment characteristics) all contributed to perceptions of positive outcomes (an intervention characteristic). In turn, such outcomes contributed to the personal satisfaction derived from delivering the intervention (a provider characteristic), increased demand for the intervention by FSWs (a client characteristic), and additional clients and enhanced reputation of the clinic (organizational characteristics). The need for ongoing technical assistance (a provider characteristic) and inducement to visit the clinic where the intervention could be delivered (a client characteristic) were both connected to the ability to provide such resources (an organizational characteristic), which, in turn, was connected to access to financial support (an external environment characteristic). This latter issue is an ongoing concern where effectiveness and implementation studies support the initial implementation but may not adequately consider long-term sustainment. However, while the results suggest several potential associations among the five sets of requirements, additional research is required to determine whether the associations are direct or indirect (i.e., whether some characteristics act as mediators for other characteristics), and the direction of causality.

Third, the findings also suggest differences in perceived requirements based on role within an organization. The percentages of counselors and clinic leaders who identified provider (85.2% versus 85.7%), client (59.2% versus 57.1%) and organizational (37.0% versus 42.9%) characteristics were quite similar to one another. However, 57.1% of leaders identified characteristics of the external requirement as a requirement for sustainment, compared to 33.3% of counselors. On the other hand, 37.0% of counselors identified evidence of positive outcomes (an intervention characteristic) as a requirement for sustainment, compared to 14.3% of leaders. Similar differences in identification of factors that could potentially impede or facilitate the adoption, implementation and sustainment of an intervention have been reported elsewhere [47] and point to the fact that perceptions that are informed by different responsibilities within the organization. For instance, clinic directors who are responsible for the financial well-being and capacity of the entire organization would be expected to be concerned with issues related to reimbursement and capacity, while counselors who are more directly involved in using the intervention and working with FSWs would be more likely to have opinions related to the effectiveness of the Mujer Segura intervention in the absence of any objective outcome data.

There are several limitations that must be kept in mind when evaluating these findings and their significance. As a qualitative investigation, the generalizability of these findings is limited by the nonrandom but purposeful selection of study participants representing directors, supervisors and counselors employed by CBOs linked to one national organization tasked with serving FSWs and other women in Mexico. However, this concern is somewhat mitigated by the fact a very high proportion of eligible clinic staff participated. The specific needs and perspectives of this stakeholder group must also be kept in mind when evaluating the significance of their descriptions of requirements for sustaining innovative programs and practices. As noted earlier, there were some differences with respect to those requirements identified by leaders and those identified by counselors. Other stakeholders, including MexFam central administration and local, state and national health officials, nonclinical staff, and clients may present additional non-overlapping requirements based on their respective roles. A more systematic survey of a random sample of each group is required to assess generalizability of these results. Further, the reliance on answers provided to specific questions during the interviews may limit the identification of requirements that may be found in other parts of the interview. The original study design called for direct payment to each site for research related activities (e.g., pay for outreach workers); however, for practical reasons funds to sites flowed from the MexFam Central Office. A cost effectiveness analysis is planned to examine how funds impacted fidelity and efficacy in the present trial. The interviews were conducted shortly after completion of the effectiveness trial, thus limiting the period during which sustainment could occur and be observed by study participants. A longer period of observation may have led to identification of additional requirements or change in emphasis of some of the requirements identified. Finally, the focus on the sustainment of an individual level intervention targeting sexual risk reduction in Mexico limits our ability to generalize to other evidence-based HIV prevention approaches in other LMICs. While the clinical focus was on improving HIV prevention for FSWs, the implementation strategy was at the clinic level. A number of sustainment factors, some unexpected, were identified as important for participating clinics, providers, and clients. Such a multi-level perspective is critical when considering the facilitation of evidence-based HIV prevention sustainment in LMICs.

## Conclusions

Despite these limitations, the findings of this study provide some insight as to the requirements for successfully sustaining individual level HIV prevention approaches in LMICs like Mexico. In addition to requiring demand for services among the populations at risk, political will to implement these programs, and continuing efforts to reach key populations year after year [2], successful implementation of current HIV prevention approaches requires provider competence in delivering the intervention, a motivation for doing so that is derived from personal experience and community impact, accessibility of both clients and providers to one another, as well as continued financial and technical support.
